# CCL2 Accelerates Microglia-Mediated Aβ Oligomer Formation and Progression of Neurocognitive Dysfunction

**DOI:** 10.1371/journal.pone.0006197

**Published:** 2009-07-10

**Authors:** Tomomi Kiyota, Masaru Yamamoto, Huangui Xiong, Mary P. Lambert, William L. Klein, Howard E. Gendelman, Richard M. Ransohoff, Tsuneya Ikezu

**Affiliations:** 1 Center for Neurovirology and Neurodegenerative Disorders, University of Nebraska Medical Center, Omaha, Nebraska, United States of America; 2 Department of Pharmacology and Experimental Neuroscience, University of Nebraska Medical Center, Omaha, Nebraska, United States of America; 3 Department of Internal Medicine, University of Nebraska Medical Center, Omaha, Nebraska, United States of America; 4 Department of Neurobiology and Physiology, Northwestern University, Evanston, Illinois, United States of America; 5 Department of Neurosciences, Lerner Research Institute, Cleveland Clinic Foundation, Cleveland, Ohio, United States of America; Mental Health Research Institute of Victoria, Australia

## Abstract

**Background:**

The linkages between neuroinflammation and Alzheimer's disease (AD) pathogenesis are well established. What is not, however, is how specific immune pathways and proteins affect the disease. To this end, we previously demonstrated that transgenic over-expression of CCL2 enhanced microgliosis and induced diffuse amyloid plaque deposition in Tg2576 mice. This rodent model of AD expresses a Swedish β-amyloid (Aβ) precursor protein mutant.

**Methodology/Principal Findings:**

We now report that CCL2 transgene expression accelerates deficits in spatial and working memory and hippocampal synaptic transmission in β-amyloid precursor protein (APP) mice as early as 2–3 months of age. This is followed by increased numbers of microglia that are seen surrounding Aβ oligomers. CCL2 does not suppress Aβ degradation. Rather, CCL2 and tumor necrosis factor-α directly facilitated Aβ uptake, intracellular Aβ oligomerization, and protein secretion.

**Conclusions/Significance:**

We posit that CCL2 facilitates Aβ oligomer formation in microglia and propose that such events accelerate memory dysfunction by affecting Aβ seeding in the brain.

## Introduction

Amyloid-β peptide (Aβ) is the principal component of cerebral amyloid deposition seen as the hallmark of Alzheimer's disease (AD). However, how plaques accumulate and how clearance is affected by microglia is poorly understood. Early ultrastructural studies suggest that microglia affect progression of cerebral amyloidosis [1]. Such defective microglial clearance functions have recently been supported by long-term *in vivo* imaging of amyloid plaques. These studies demonstrated long-term stability of amyloid deposits following Aβ synthesis interruption by the Tet-off system. This extravascular protein synthesis progresses even though microglia are in close proximity to the newly developed plaques [2], [3]. Age-related microglial dysfunction for amyloid clearance also correlates with enhanced expression of pro-inflammatory cytokines and reduced phagocytosis [4]. In contrast, recruitment of peripheral blood-borne macrophages into the brain parenchyma dramatically enhances amyloid clearance, suggesting the possibilities of contrasting roles of resident microglia and recruited blood monocyte-derived perivascular macrophages for amyloid clearance [5], [6].

Monocyte chemotactic protein-1 (MCP-1/CCL2) is a β-chemokine responsible, in part, for the chemotaxis of mononuclear phagocytes (MP; microglia, peripheral monocytes and macrophages). CCL2 levels in the cerebrospinal fluid and sera are linked to neurodegeneration. Indeed, the amount of CCL2 shows a negative correlation with cognitive scores in mild cognitively impaired (MCI) and AD patients [7]. These findings suggest that elevated CCL2 is a very early event in AD pathogenesis [8]. In order to elucidate the role of CCL2 in AD pathogenesis, we developed APP/CCL2 mice [9]. These mice were made by crossing an established Aβ deposition mouse model (Tg2576) with a CCL2 over-expressing mouse under the regulation of glial acidic fibrillar protein (GFAP) promoter (JE-95) [10]. CCL2 over-expression led to microgliosis and increased diffuse plaque formation in APP/CCL2 bigenic mice. Interestingly, both increased CCL2 signaling in our APP/CCL2 bigenic mice and deficient CCL2 signaling in APP/CCR2^−/−^ mice worsened AD pathology in different manners [11]. We now posit that intrathecal CCL2 expression (as observed in AD) accelerates beta-amyloidosis. By contrast, circulating ‘inflammatory’ (Ly6-C^hi^/CCR2^+^) monocytes affect Aβ clearance in APP transgenic mice.

To address this apparent discrepancy we have now extended our studies in two ways: (1) we evaluated hippocampal neurophysiology, memory and cognition to establish the functional importance of the neuropathologic endpoints; and (2) we assessed whether microglia affect conversion of monomeric Aβ to oligomer form, a key step towards cognitive dysfunction and subsequent fibril formation [12], [13]. Accelerated neurodegeneration was found in APP/CCL2 bigenic mice. Our findings are highly relevant to AD pathogenesis, and direct inhibition of CCL2 signaling will reduce microglial activation in a fashion that will both lower Aβ deposition and improve behavioral outcomes. Suppressing CCR2 function, by contrast, can exert an opposite effect by impairing Aβ metabolism and disease.

## Results

### Accelerated memory impairments in APP/CCL2 mice

**Figure 1:**
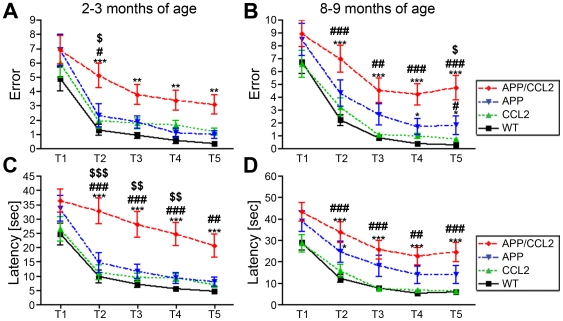
Accelerated spatial learning impairment in APP/CCL2 mice. The same animals were tested by RAWM test at two different time points: 2–3 (A and C) and 8–9 months of age (B and D). The numbers of animals were 9 APP/CCL2, 10 CCL2, 8 APP, and 10 WT mice (all littermates from cross breeding of APP and CCL2 hemizygotes). The compiled average errors (A and B) and latency (C and D) of Trial 1-5 are shown. *, ** or *** denotes p<0.05, 0.01 or 0.001 versus WT, #, ## or ### denotes p<0.05, 0.01 or 0.001 versus CCL2, and $, $$ or $$$ denotes p<0.05, 0.01 or 0.001 versus APP group, respectively, as determined by two-way ANOVA and Bonferroni posttests.

Tg2576 mice exhibit impaired memory retention and memory acquisition by 6 and 12 months of age, respectively, when tested by hidden platform or by a radial arm water maze (RAWM) test [14]. A10-day RAWM task was employed to assess working (short-term) memory in APP/CCL2, CCL2, APP, and WT mice at 2–3 and 8–9 months of age but using the same mice. RAWM has been used effectively with mice to measure hippocampal function [14], [15]. In this learning test paradigm, an average error number of less than one by trial 4 (T4) or T5 is regarded as secured memory formation or recall, respectively. All animal groups, including APP mice, showed secured memory formation and recall except the APP/CCL2 bigenic mice, at 2–3 months of age, when errors at T4 and T5 were assessed (Fig. 1A). Average latency (or time to reach the correct platform) was used, in parallel tests, to measure memory formation and recall. As a reference, the average latencies at T4 and T5 were similar among all groups with the exception of a significantly longer latency in APP/CCL2 mice (Fig. 1C). APP and CCL2 mice have normal memory function at 2–3 months of age, whereas APP/CCL2 mice have already developed memory impairment. Consistent with previous reports, APP mice develop memory impairments by 8–9 months when compared to age-matched WT or CCL2 littermates (T4 and T5, Fig. 1B). However, APP/CCL2 mice show more severe memory impairments as determined by errors and latencies at T4 and T5, when compared to APP, CCL2, or WT mice (Fig. 1B, D). These data suggest that memory formation and retention were more significantly impaired in APP/CCL2 than in APP mice as early as 2–3 months and were sustained to 8–9 months. To rule out impairment of vision, motor coordination, or motivation as causes for defective memory formation we tested the same mice for balance beam behavior, body weight, and voluntary swimming speed in the open field at two different ages (Supporting information (SI) Fig. S1). The numbers of crossed segments on the balance beam, indicative of basic sensory-motor coordination, were significantly higher in CCL2 groups when compared to the 8–9 months age groups. Some differences in voluntary activity of animals among groups were seen but did not correlate with the RAWM results. Lower body weights were observed in the APP mice when compared to WT animal groups at 2–3 months. Such weight differences were also observed in the APP/CCL2 and APP groups when compared to CCL2 mice at 8–9 months. Nonetheless, voluntary swimming speeds of APP/CCL2 were similar to WT and CCL2. APP mice swam faster than the WT group at 8–9 months excluding the possibility that a lower body weight resulted in slower swimming speeds. Overall, these data support the idea that learning and memory formation do not affect swimming or locomotor function at any mouse age. Thus, impaired working memory, measured by errors and latency, was readily seen in APP/CCL2 mice.

### Synaptic dysfunction in APP/CCL2 hippocampus

**Figure 2:**
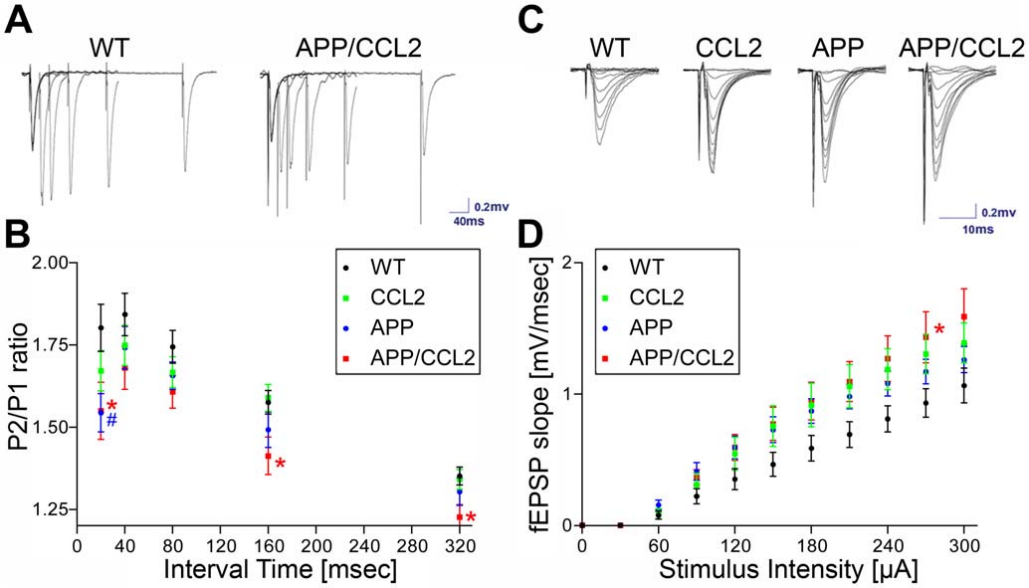
Paired-pulse facilitation and input-output responses recorded in the CA1 region of mouse hippocampal slices. WT (n = 15 slices from 8 animals), CCL2 (n = 13 slices from 7 animals), APP (n = 12 slices from 7 animals) and APP/CCL2 mice (n = 14 slices from 7 animals) at 5–6 months of age were tested. (A) Superimposed sample traces of field excitatory postsynaptic potentials (fEPSPs) recorded in the CA dendritic field in response to twin pulse stimulation of Schafer-collateral pathway at various interpulse intervals (as shown in B) in WT and APP/CCL2 mice. (B) Graph plots of averaged paired-pulse facilitation (PPF) ratio at 20-, 40-, 80-, 160-, and 320-msec interpulse intervals in 5–6-month-old WT, CCL2, APP and APP/CCL2 mice. A significant difference between WT and APP/CCL2 mice (*p<0.05) was found at 20-, 160-, and 320-msec intervals and WT versus APP (#p<0.05) at 20-msec intervals as determined by ANOVA and Newman-Keuls *post hoc* (p<0.05). (C) Representative traces of superimposed fEPSPs, evoked by single pulse stimulations at various intensities ranging from 30 to 300 µA with an increment of 30 µA, were recorded from slices of WT, CCL2, APP and APP/CCL2 mice, respectively. (D) Averaged input-output curves generated using different stimulus intensities ranging from 0–300 µA in 30 µA increments as shown in C. Significant difference between WT and APP/CCL2 (*p<0.05) at the stimulus intensity of 270 µA was determined by ANOVA and Newman-Keuls *post hoc*.

Previous studies demonstrated that APP mice display abnormalities in synaptic transmission and long-term potentiation (LTP) [16], [17], [18], [19]. Based on these observations, synaptic function in WT and transgenic mice were measured to determine if there was any functional impairment in the hippocampal CA1 region. Paired-pulse facilitation (PPF), a measure of short-term synaptic plasticity, was examined in 5–6-month-old WT, CCL2, APP, and APP/CCL2 mice. These animals were not used in behavioral analysis in order to minimize potential complications due to maze training. PPF curves were generated at interpulse intervals of 20, 40, 80, 160, and 320 msec. Significant impairments were observed in PPF at 20-, 160-, and 320-msec intervals in APP/CCL2 and at 20 msec in APP compared to WT mice (Fig. 2B). These results support the notion that decreased presynaptic neurotransmitter release is operative during the second stimulus in APP/CCL2 mice.

To investigate basal synaptic transmission, input-output curves for field excitatory postsynaptic potentials (fEPSP) were measured in the CA1 region of the hippocampus. WT mice showed smaller fEPSP slopes at all stimulus intensities (Fig. 2D). The average slopes of fEPSP at 270 mA stimulus intensity in WT and APP/CCL2 mice were 0.93±0.1 mv/msec (n = 15 slices from 8 animals) and 1.43±0.19 mv/msec (n = 13 slices from 7 animals; p<0.05), respectively. These data indicate that basal synaptic transmission is enhanced in APP/CCL2 mice at 5–6 months of age, suggesting the upregulation of postsynaptic neurotransmitter receptors to compensate decreased presynaptic neurotransmitter release as reported [20] [21].

### APP/CCL2 mice show enhanced Aβ deposition and oligomer formation

**Figure 3:**
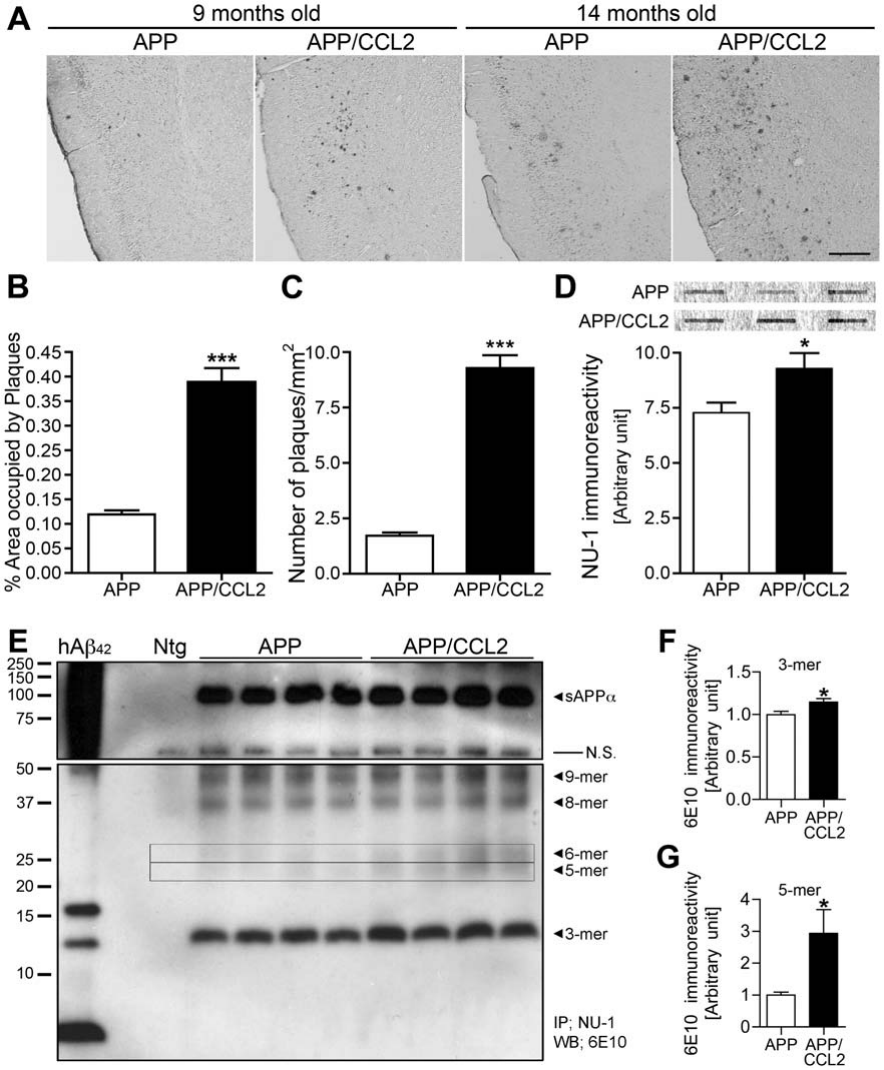
Enhanced Aβ oligomer formation and deposition in APP/CCL2 mice. (A) Frozen sections (10-µm thickness) of temporal cortex of APP and APP/CCL2 mice at 9 and 14 months of age were immunostained by Aβ antibody and visualized by DAB staining. Scale bar, 400 µm. (B and C) Quantification of cortical Aβ staining in APP and APP/CCL2 mice at 9 months of age (n = 5 per group). Percent area occupied by immunoreactivity (C) and numbers of plaques were measured. *** denotes p<0.001 vs APP/CCL2 as determined by Student's *t*-test. (D) APP/CCL2 and APP mice were sacrificed at 9 months of age and 2% SDS-soluble fractions were subjected to dot blot assay for Aβ oligomer using NU-1 as described (each brain sample per dot). Top panel is a representative dot blot image (3 representative dots per group out of n = 6 per group). Bottom panel shows quantification of band luminescent intensities (n = 6 per group). * denotes p<0.05 vs APP/CCL2 as determined by Student's *t*-test. (E–G) Identification of Aβ oligomers in the brains of APP and APP/CCL2 mice assessed by immunoprecipitation of Aβ oligomers from extracellular-enriched fraction using NU-1 anti-Aβ oligomer monoclonal antibody and Western blot using biotinylated 6E10 anti-Aβ monoclonal antibody. Oligomerized synthetic human Aβ1–42 peptide was used as a size marker and positive control of Aβ oligomers (hAβ42, left lane). Arrows indicate respective migration positions of trimers (3-mer), pentamer (5-mer in box), hexamers (6-mer in box), octamer (8-mer), and nonamers (9-mer). Band luminescent intensities for trimers (F) and pentamers (G) were quantified by Image J software. * or *** denotes p<0.05 or 0.001 as determined by Student's *t*-test, respectively.

We examined the Aβ load in APP/CCL2 mice. As expected, enhanced Aβ deposition was seen in bigenic APP/CCL2 mice when compared to APP mice at 9 and 14 months, and reflective of our previous work (Fig. 3A–C, [9]). SDS-stable Aβ oligomers are responsible for LTP abnormalities and precede Aβ deposition [22]. This is followed by Aβ-induced neurotoxicity, hippocampal degeneration [23], and memory impairment in Tg2576 [13]. Aβ oligomers from Tg2576 or AD brains are linked to memory impairments and synaptic loss [13], [24]. Based on this information, we examined whether Aβ oligomers are increased in APP/CCL2 mice. This was assayed by dot blot analysis of ultracentrifuged lysates using specific Aβ oligomer antibodies (clones NU-1 [25]). APP/CCL2 bigenic mice showed increased Aβ oligomer accumulation when compared to age-matched APP mice (Fig. 3D). This was confirmed by Aβ immunoblotting of the extracellular-enriched fraction of brain lysates (Fig. 3E–G). The amounts of hexamer (6-mer), octamer (8-mer) and nonamer (9-mer) were increased in APP/CCL2 bigenic mice but did not reach significance (data not shown). The amounts of trimer (3-mer) and pentamer (5-mer) were increased in APP/CCL2 bigenic mice compared to APP mice (Fig. 3F, G). These data suggest that the early onset of memory impairment is due to enhanced accumulation of Aβ oligomers in APP/CCL2 bigenic mouse brains.

**Figure 4:**
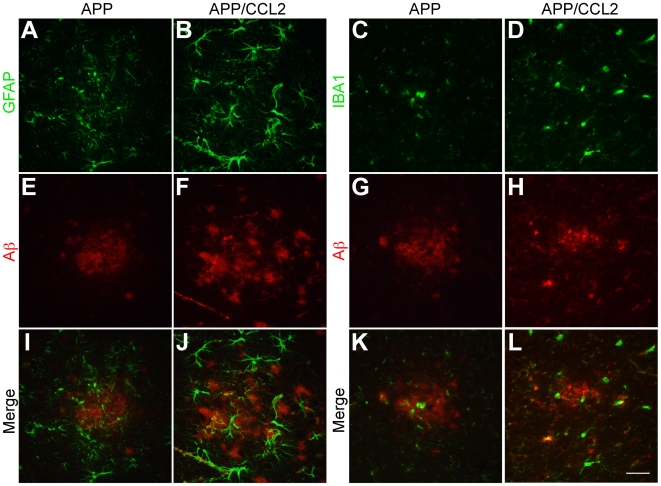
Accumulation of astrocytes and microglia to Aβ deposits. Frozen sections (10-µm thickness) of temporal cortex of APP and APP/CCL2 mice at 9 months of age were subjected to double immunofluorescence for GFAP (A–B, green) or IBA1 (C–D, green) and Aβ (NU-1) (E-H, red). (I–L) Merged images. Scale bar, 50 µm.

CCL2 levels in the hippocampus of each of the transgenic groups were analyzed. A 360 to 800-fold increase in levels of CCL2 protein was observed in the hippocampus of APP/CCL2 and CCL2 mice (36.0 and 47.5 ng protein/mg brain protein, respectively) when compared to APP and WT mice (99.9 and 58.9 pg/mg, respectively; SI Fig. S2). Aβ deposits are associated with astro- and micro- gliosis, which was enhanced in APP/CCL2 bigenic mouse brains compared to APP mice (Fig. 4A–L). This suggested that CCL2 affects Aβ oligomer formation in areas adjacent to microglia accumulation.

### CCL2 and tumor necrosis factor (TNF-α) enhance intracellular Aβ microglial oligomer formation

**Figure 5:**
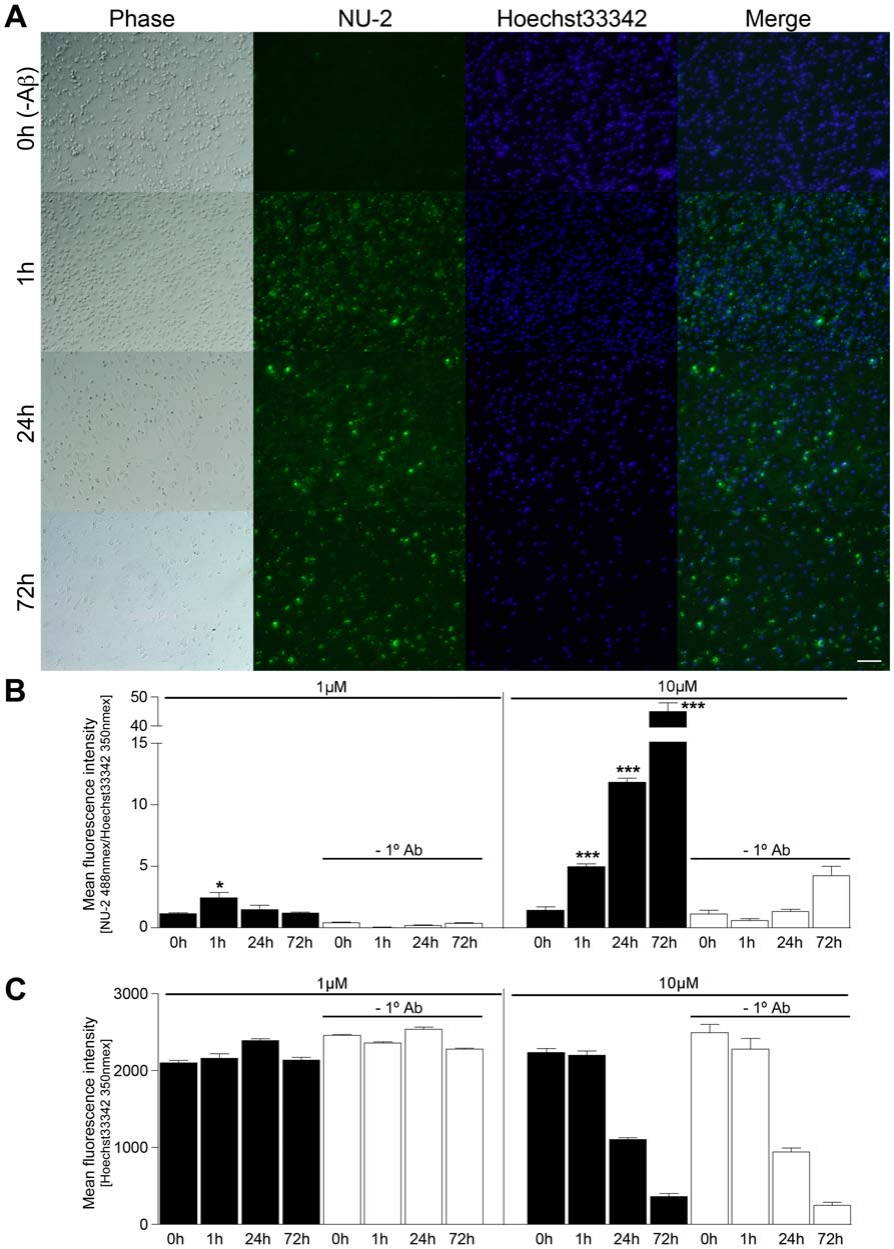
Aβ aggregation in mouse primary microglia. Mouse primary microglia (5×10^4^ per well) were seeded in 96-well tissue culture plates, and incubated with monomeric Aβ42 (1 or 10 µM) in phenol red-free DMEM for 0 (-Aβ), 1, 24, and 72 h, followed by washing, fixation, and immunocytochemistry with NU-2 anti-Aβ oligomer antibody (green) and Hoechst 33342 for nuclear staining (blue). (A) Representative phase and merged immunofluorescet images after incubation with 10 µM Aβ42. Scale bar, 100 µm. (B, C) Fluorescent intensity of Aβ (B) or nuclear (C) signals were quantified using fluorometer (Ex/Em 488 nm/519 nm for Alexa 488, and 350 nm/461 nm for Hoechst 33342, respectively, n = 3 per group). −1° Ab represents negative control staining of microglia for Aβ (B) or nuclear (C) staining using secondary antibody (Alexa 488 anti-mouse IgG) and Hoeschst 33342 for nuclear staining. * or *** denotes p<0.05 or 0.001 versus 0 h incubation, respectively.

To determine the effect of brain CCL2 expression on Aβ aggregation, we tested Aβ production, degradation and aggregation. Our prior works demonstrated that CCL2 does not affect APP neuronal expression and Aβ production, nor does it reduce intracellular Aβ degradation in bone marrow derived macrophages *in vitro* or in APP/CCL2 bigenic mice *in vivo* [9], [26]. Thus, we examined if Aβ aggregation, especially oligomer formation, is enhanced after phagocytosis of monomeric Aβ42 in microglia. Here a new system to assay cell Aβ aggregation was developed by combinations of microglial culture with immunofluorescence and fluorometry using 96-well tissue culture plates (see Materials and Methods). To optimize incubation time and Aβ concentration, primary cultured mouse microglia were incubated with monomeric Aβ42 at 1 or 10 µM for 0, 1, 24 and 72 h, followed by immunofluorescence of aggregated Aβ with Aβ oligomer antibodies (NU-2) and nuclear staining (Hoechst 3342). Quantification of the fluorescent signals was made by fluorometry of the immunostained cells in 96-well plates. We observed rapid Aβ oligomer formation in 1 h after the uptake of monomeric Aβ42 in microglia, and the intensity of Aβ increased at 24 and 72 h for 10 µM Aβ42 (231.7% and 909.4% increase over 1 h time point), although the intensity decreased at 24 and 72 h for 1 µM Aβ42 (61.3% and 48.7% decrease over 1 h time point) (Fig. 5A–B). However, the number of nuclei was significantly reduced at 24 and 72 h after the administration of 10 µM Aβ42 (50.6 and 83.7% reduction as compared to the 0 h time point, respectively). We posit that this was most likely caused by the cytotoxicity at high concentrations of Aβ42 (Fig. 5C). However, 1 h incubation of 10 µM Aβ42 showed sufficient Aβ oligomer immunofluorescent intensity without cytotoxicity (Fig. 5B–C). Thus, a 1 h assay was performed at 10 µM of Aβ42.

**Figure 6:**
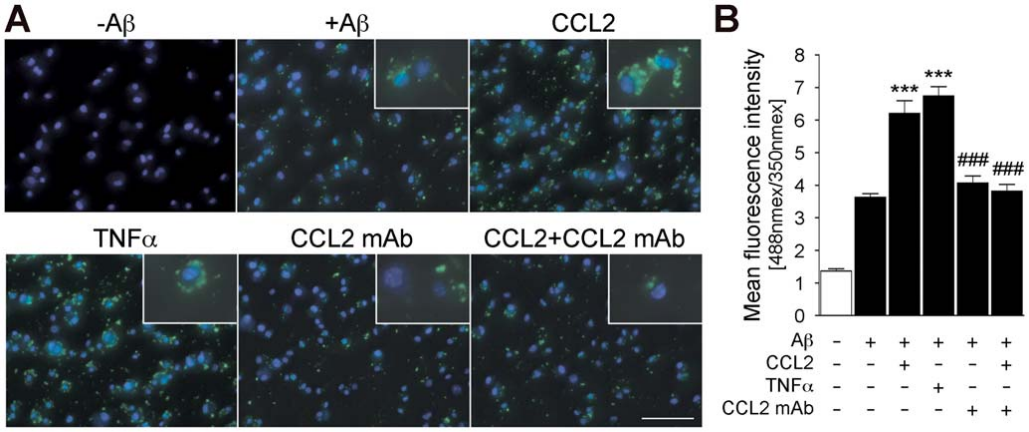
CCL2 and TNF-α enhances Aβ aggregation in mouse microglia. (A) Primary mouse microglia were incubated without Aβ (-Aβ), with Aβ (other panels), and co-incubated with 10 ng/ml CCL2, with 10 ng/ml TNF-α, with 1 µg/ml neutralizing anti-CCL2 antibody (CCL2 mAb), or co-incubated with 10 ng/ml CCL2 and 1 µg/ml CCL2 mAb (CCL2+CCL2 mAb) for 1 h, followed by immunofluorescence with NU-2 anti-Aβ oligomer antibody (green) and Hoechst33342 for nuclear staining (blue). Merged captured images were shown. Insets are high-magnification. Scale bar, 100 µm. (B) Flurolometric quantification of Aβ oligomer fluorescent signal (Ex/Em = 488 nm/519 nm) normalized by the nuclear staining signal (Ex/Em = 350 nm/461 nm, n = 3 per group). White or black bars represent incubation of microglia with no Aβ (white, negative control) or with 10 µM Aβ42 (black) Aβ, respectively. *** and ### denote p<0.001 versus Aβ group, and versus CCL2 group, respectively.

**Figure 7:**
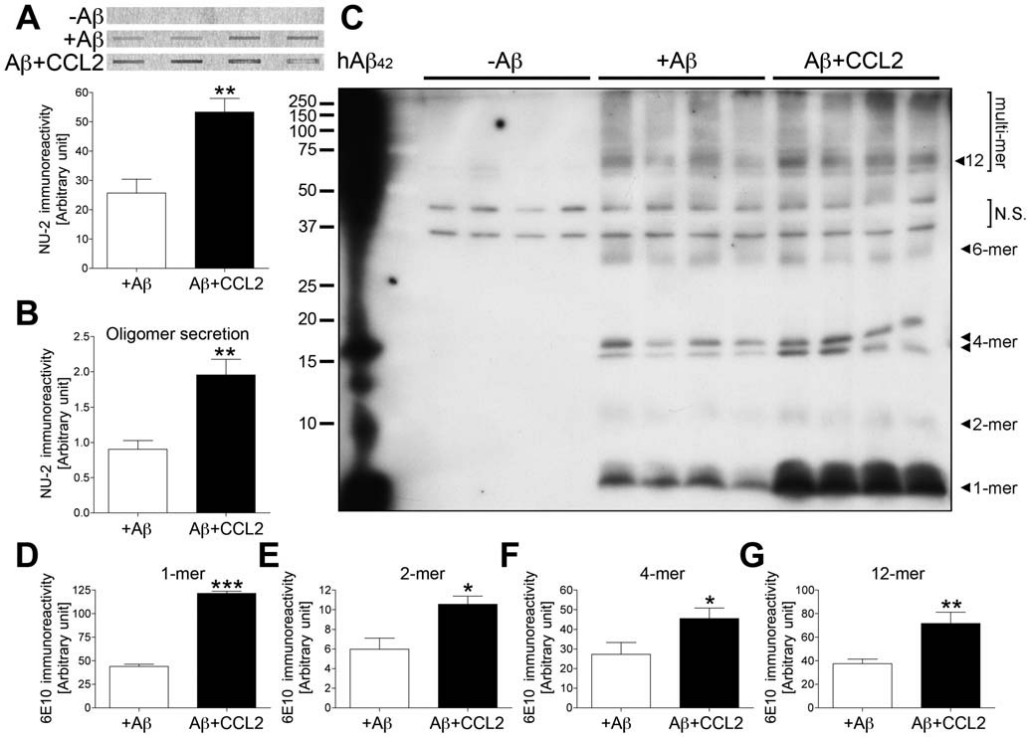
CCL2 accelerates intracellular Aβ microglial oligomer formation. (A, B) Quantification of Aβ oligomers in mouse microglia (A), or secreted from mouse microglia (B), incubated without Aβ (-Aβ), with Aβ (+Aβ), and co-incubated with CCL2 (10 ng/ml, Aβ+CCL2) for 1 h, assessed by dot blot. Band luminescent intensities were quantified by Typhoon Phosphoimager. ** denotes p<0.01 as determined by Student's *t*-test. (C) Identification of Aβ oligomers in mouse microglia assessed by Western blot. Synthetic human Aβ1–42 peptide (hAβ42) was used as a size marker and positive control (left lane). Arrows indicate respective migration positions of monomers (1-mer), dimers (2-mer), tetramers (4-mer), hexamers (6-mer) and dodecamers (12-mer). Band luminescent intensities for monomers (D), dimers (E), tetramers (F), and dodecamers (G) were quantified by Image J software. *, **, or *** denotes p<0.05, 0.01, or 0.001 as determined by Student's *t*-test, respectively.

Next, we examined the effect of CCL2 in microglia. As shown in Fig. 6A–B, we observed rapid Aβ oligomer formation 1 h after the uptake of 10 µM monomeric Aβ42 in microglia, which is significantly enhanced by co-incubation with CCL2 or TNF-α. Inactivation of endogenous or recombinant CCL2 using a neutralizing antibody against CCL2 (CCL2 mAb) significantly reduced oligomer formation (Fig. 6B), demonstrating its specific induction through the CCL2 receptor. These findings were confirmed by immunoblot analysis of cell lysate by NU-1 dotblot assays (Fig. 7A) and 6E10 immunoblottings (Fig. 7C). We found that the amount of monomer, dimer, tetramer, and 12-mer was increased in CCL2-treated microglia as compared to control, whereas the hexamer level was found unchanged (Fig. 7D–G). In addition, secretion of Aβ oligomer from Aβ-phagocytized microglia was also enhanced by CCL2 (Fig. 7B), demonstrating that microglia can be a source of Aβ oligomer formation. This is consistent with the fact that a certain fraction of phagocytized Aβ aggregates can be secreted into extracellular fluid from primary microglia, bone marrow- or monocyte-derived macrophages [26], [27], [28]. The data, taken together, suggest that Aβ aggregates can be secreted and serve as Aβ seeds for further plaque formations. The entire process is enhanced by CCL2.

## Discussion

We demonstrated that CCL2 is a potent enhancer of Aβ oligomerization, microglial accumulation, and cognitive dysfunction in an animal model of beta-amyloidosis. These findings are secondary to Aβ aggregation as over-expression of CCL2 alone has no effect on cognitive function. The data support the idea that CCL2 is a co-factor in Aβ-induced memory impairment for APP mice. The effect of Aβ with CCL2 co-expression was also observed for spatial learning, PPF, and input-output responses. PPF is a measure of presynaptic function, thought to result from an increase in transmitter release due to a calcium influx into the presynaptic terminal during responses to an initial stimulus. Although PPF is not a direct measure of the transmitter release from the presynaptic terminals, the impairment of PPF suggests that the presynaptic terminals in APP/CCL2 mice may be less able to sustain neurotransmitter release with repetitive stimulation. Moreover, significant enhancement of the input-output responses in APP/CCL2 mice was observed at 270 mA stimulus intensities when compared to WT mice. The enhancements of input-output responses in APP/CCL2 could explain why Aβ induces altered intracellular calcium levels and membrane excitability by blocking fast-inactivating potassium (A) currents [29]. We have also examined the hippocampal CA1 LTP evoked by tetanic stimulation of the Schaffer collateral pathway using APP/CCL2, APP, CCL2, and WT mice at 6 months of age, which did not affect LTP impairments (data not shown). This suggests that the effect of endogenously developed Aβ oligomers is not sufficient to induce LTP impairments and that 10-day RAWM tests are a more sensitive measure to assay neuronal dysfunction.

Our previous study on APP/CCL2 mice demonstrated that microglial accumulation enhances diffuse plaque formation, suggesting the enhancement of Aβ aggregation by CCL2. Indeed, Aβ oligomers are quantitatively elevated in APP/CCL2 mice as compared to age-matched APP littermates in this study. Since Aβ oligomer levels directly correlate with memory impairment, it is likely that CCL2 accelerates the onset of memory impairment by enhancement of Aβ oligomerization in the brain. However, extracellular Aβ oligomers in APP or APP/CCL2 bigenic mice were undetectable using our system at 2–3 months of age (data not shown). This suggests that very low amounts of Aβ oligomer could induce memory dysfunction in young mice, or, alternatively, a combination of CCL2 and other inflammatory factors elicited by Aβ production (such as reactive oxygen species) may influence memory formation. Indeed, Aβ oligomers inhibit LTP induction partly through microglial activation and inducible nitric oxide synthase and superoxide formation [30]. Although the exact mechanism behind how CCL2 enhances Aβ oligomerization is unknown, our data demonstrates that Aβ uptake by microglia was significantly enhanced by CCL2, which resulted in an increase of oligomerized Aβ species in both intracellular and extracellular fractions. Microglia mediate Aβ internalization through a nonsaturable, fluid phase macropinocytic mechanism. This mechanism is distinct from phagocytosis and receptor-mediated endocytosis [31] and dependent on actin and tubulin dynamics. Thus, it is possible that CCL2, which is a well-known chemoattractant protein for microglia, may prominently activate cytoskeletal dynamics as a part of its chemotactic signaling and coincidentally affect Aβ uptake. It should be noted that the NU-2 staining of microglia is 70% preserved without cell permeabilization (data not shown). This suggests that majority of the Aβ aggregation seems to take place on the cell surface rather than intracellularly. This is also consistent with the fact that CCL2 rather suppresses Aβ-stimulated microglial phagocytosis [32]. Altogether, it is possible that CCL2 mobilizes actin and tubulin cytoskeletal dynamics, which enhance Aβ uptake. In this manner, cell surface Aβ can rapidly aggregate to form Aβ oligomers. We have previously shown that Aβ oligomer uptake was strikingly negligible as compared to those of monomeric or fibrillar Aβ in monocyte-derived macrophages [28]. This suggests that oligomeric Aβ will dramatically lose its binding affinity to the membrane and be secreted. This may be a mechanism for CCL2-mediated Aβ oligomer formation and Aβ seeding for developing daughter plaques surrounding the parent amyloid plaques.

We did not observe significant microgliosis in the hippocampus prior to Aβ deposition in the APP or APP/CCL2 mouse brain as compared to non-Tg littermates, although we did see accumulation of microglia surrounding either diffuse or compact plaques. This is in contrast to the report of the APP/CCR2^−/−^ mouse study, which shows enhanced microgliosis in APP mice prior to the parenchymal Aβ deposition [11]. However, APP/CCR2^−/−^ mice develop accelerated amyloid angiopathy and shortened lifespans. These data suggest the contrasting role of CCL2 to microglia and peripheral monocytes. In brain parenchyma, CCL2 may enhance microgliosis around amyloid depositions and enhance beta-amyloidosis partly through Aβ oligomer formation in microglia. In periphery, CCL2 is a key molecule for the CNS recruitment of circulating monocytes, which are 7/4^bri^Ly-6G^−^CCR2^+^F4/80^+^ [33] or Ly-6C^hi^CCR2^+^ [34], and play a critical role in amyloid clearance [5]. Disruption of CCR2 leads to impaired mobilization of monocytes from bone marrow and their CNS recruitment, which will ultimately led to enhanced beta-amyloidosis. Thus while brain CCL2 production may enhance beta-amyloidosis, peripheral CCL2 will enhance clearance.

Neurodegeneration found in young APP/CCR2^−/−^ mice suggests the impaired neuroprotective effect of brain microglia or recruited monocytes by disruption of CCR2. Although resting microglia are neuroprotective, upon stimulation of innate immunity mechanisms by a myriad of molecules, such as pro-inflammatory cytokines and Toll-like receptor ligands, microglia convert to an inflammatory phenotype and become neurotoxic. In accord, microglia in APP mice show pro-inflammatory activation by aging [4]. Thus, suppression of CCL2 signaling in aged brains may not develop adverse effects on neurons. Interestingly, CCR2^−/−^ mice showed enhanced noise-induced inner hair cell loss, independent of CCL2 signaling, suggesting CCL2-independent neuroprotective responses [35]. Thus, neutralization of CCL2 in CNS should not affect the neuroprotective signaling of CCR2 for therapeutic invention, and CNS suppression of chemokine-mediated microgliosis through chemokine inhibitors may be of therapeutic interest. Other chemokines, such as IL-8, upregulated in MCI and AD patients, are likely to have similar functions in microgliosis and cognitive dysfunction for APP mice. The functions of these other chemokines need to be a focus of future investigations. The cumulative prediction was that increased intrathecal CCL2 would be deleterious for APP transgenic mice. Further study will be necessary to determine how blockage of brain CCL2 affects AD progression in affected people.

## Materials and Methods

### Animals

All the animal studies followed guidelines and were approved by the Institutional Animal Care and Use Committee at University of Nebraska Medical Center. The transgenic mice used in this study were described previously [9]. Briefly, Tg2576 mice expressing the Swedish mutation of human APP_695_ were obtained from Drs. G. Carlson and K. Hsiao-Ashe through Mayo Medical Venture [36]. Both Tg2576 mice and CCL2 (JE-95 strain) mice were backcrossed at least four times to 129/Sve mice to develop a 129/Sve strain. Male Tg2576 were crossed with CCL2 females to generate WT (APP and CCL2 transgene negative), CCL2 (APP transgene negative and CCL2 transgene positive), APP (APP transgene positive and CCL2 transgene negative), and APP/CCL2 (APP and CCL2 transgene positive) mice. The APP, APP/CCL2, WT, and CCL2 mice were littermates.

### Genotyping

DNA samples were prepared from cut tail tips (<1.0 cm) of individual pups, and genomic DNA was extracted using the Easy DNA kit (Invitrogen). PCR was performed as described previously to identify APP transgene-positive mice [37]. The human *GFAP* promoter murine *CCL2* transgene was confirmed by PCR of genomic DNA with primers: JE-5 (5′-TTC CTG GGC ACA GGC TGA ATA GAG) and JE-3 (5′-ATT GAG CAG GGG GCT TGC ATT G), which amplify a 150 bp DNA fragment from the transgene, but not the endogenous murine CCL2 gene as described [9], [10].

### Weight, balance beam, and swimming speed monitoring

Weight was measured, balance beam test was performed and the swimming speed was determined for all the mice run through the 10-day RAWM test. For the balance beam task, each animal was placed at the center of a suspended beam that is segmented at 10 cm intervals (100 cm in length) and released as described [14]. Whether the mice fell off the platform and the number of segments crossed for each trial (3 min) was recorded. For subsequent analysis of swimming speed the inserts were removed from the tank used for RAWM and tracking software (PolyTrack, San Diego Instruments) recorded the path of the rodent by light/dark contrast detection by a video camera (Burle Securities) suspended 170 cm above the liquid surface as described [38].

### Ten-day RAWM test

Mice at 2–3 and 8–9 months of age (9 APP/CCL2, 10 CCL2, 8 APP, and 10 WT mice) were introduced into the perimeter of a circular water-filled tank 91 cm in diameter and 110 cm in height with triangular inserts placed in the tank to produce six swim paths radiating out from a central area. Spatial cues for the mice to correctly orient themselves were present on the walls of the tank. At the end of one arm a 10 cm circular hidden plexiglass platform was placed, submerged 1 cm deep. The platform was located in the same arm for 4 consecutive acquisition trials (T1 through T4), and in a different arm on different days. For T1-T4, the mouse started the task from a different randomly chosen arm, excluding the arm with the platform. Each trial lasted 1 min and an error was scored each time when the body of the mouse, excluding tail, entered the wrong arm, entered the arm with the platform but did not climb on it, or did not make a choice for 15 sec. After T1 through T4 the mouse was returned to its cage for 30 min, and then administered a retention trial (T5). For the retention trial the start arm was the same as in T4. Each trial ended when the mouse climbed onto and remained on the hidden platform for 10 sec. The time taken by the mouse to reach the platform was recorded as its latency. If the mouse did not reach the platform, 60 sec was recorded as its latency and the mouse was gently guided to the submerged platform. The mouse was given 20 sec to rest on the platform between each trial from T1 through T4. Testing was considered complete when WT mice reached asymptotic performance (one error on T4 and T5; 10 training days). The errors for the last 3 days of testing of each mouse were averaged and used for statistical analysis.

### Hippocampal slices and electrophysiology

Five to six-month-old transgenic and WT mice were anaesthetized with isoflurane, decapitated, and their brains quickly removed from the cranial cavity. The brains were placed into an ice-cold (4°C) oxygenated artificial cerebrospinal fluid (ACSF) environment. The hippocampi were dissected free and 400 µm-thick (transverse section in hippocampus) slices were cut using a tissue chopper. The slices were stored in a humidified/oxygenated holding chamber at room temperature for at least 1 h before being transferred into a recording chamber. In the recording chamber, single hippocampal slices were fully submerged in a continuously perfused ACSF solution at a constant flow rate of 2 ml/min with the use of a peristaltic pump (Rainin Instruments). The ACSF contained (in mM): NaCl (124.0), KCl (3.0), CaCl_2_ (2.0), MgCl_2_ (2.0), NaH_2_PO_4_ (1.25), NaHCO_3_ (26.0) and glucose (10.0). ACSF was equilibrated with 95% O_2_ and 5% CO_2_ and had a pH of 7.4–7.5. The temperature of the perfusion was maintained at 30±1°C with an automatic temperature controller (Warner Instruments).

Field excitatory post-synaptic potentials (fEPSPs) were generated by test pulses applied every 20 sec (low frequency) with a constant current stimulation (100–300 µA, 40 µsec in duration) of Schaffer collateral-commissural axons using an insulated bipolar tungsten electrode (except for the tip). The intensity and duration of stimulation were adjusted to generate approximately 30–40% of a maximal response. The evoked fEPSPs were recorded with an Axopatch-1D amplifier (Axon Instruments, Inc.) in the CA1 dendrite field. The recording microelectrodes were made from borosilicate glass capillaries with inner filaments that enable quick back-filling as described [39], [40]. The tip diameter of the microelectrode was about 5.0 µm and had a resistance of 1–5 MΩ when filled with ACSF.

Input-output tests were conducted using fixed pulse duration of 40 µsec with stimulation intensities increasing from 30 to 300 µA in increments of 30 µA. Paired-pulse facilitation (PPF) was assessed with twin pulses at a fixed pulse duration of 40 µsec and a varying interpulse interval (20, 40, 80, 160, 320 msec). The paired pulses were delivered at 20-sec intervals and six consecutive responses were averaged for each PPF test. The degree of facilitation was determined as the increase in ratio of the initial slope of the second response over first response in each pair.

### Protein extraction and ELISA

CCL2 ELISA was performed as described previously [9], [27]. Briefly, brain tissues were homogenized in solubilization buffer (50 mM Tris-HCl (pH 7.5), 100 mM NaCl, 2 mM EDTA-Na, 1% Triton X-100, and protease inhibitor cocktails (Roche Applied Science)) and supernatant was subjected to CCL2 ELISA (Mouse JE/CCL2, R&D System) [41].

### Dot blot

Dot blot analysis for Aβ oligomer was performed as described previously [42] with some modifications. Briefly, mouse brains were homogenized in 2% SDS containing phenylmethylsulfonyl fluoride (PMSF) (0.5 mM), leupeptin (3 µg/ml) and aprotinin (5 µg/ml), and centrifuged at 4°C for 1 h at 100,000 x *g*. Primary mouse microglia were lysed with lysis buffer containing 50 mM Tris-HCl (pH 7.6), 0.01% NP-40, 150 mM NaCl, 2 mM EDTA, 0.1% SDS, 0.1% Triton X-100, 1 mM PMSF, Leupeptin (3 µg/ml) and Aprotinin (5 µg/ml), incubate 4°C for 1 h, and centrifuged at 4°C for 10 min at 14,000 x g. Protein concentration of the supernatant was determined using BCA Protein Assay Kit (Pierce). Samples were adjusted with 1× PBS to the same concentration, and 2 µg (brain) and 10 µg (microglia) of the lysates were applied to a nitrocellulose membrane using Bio-Dot® SF Microfiltration Apparatus according to manufacture's instruction (Bio-Rad). After blotting, the membrane was incubated in 1× PBS at 97°C for 10 min, washed in Tris-buffered saline (TBS, pH 7.2) twice, then incubated in a 5% solution of nonfat dry milk for 1 h at room temperature. After overnight incubation at 4°C with anti-Ab oligomer antibody (clone NU-1; 1 µg/ml) [25], the membrane was washed in Tween 20-TBS (TTBS) (0.05% Tween 20, 100 mM Tris-HCl (pH 7.5), 150 nM NaCl) for 3×10 min and incubated at room temperature with the HRP-conjugated anti-mouse IgG (Jackson ImmunoResearch Laboratories) for 1 h. The membrane was washed in TTBS for 3×10 min and incubated for 5 min with a chemiluminescent substrate (ECL Plus, GE Healthcare Bio-Sciences). Images were taken using an image analyzer (Typhoon, Amersham Biosciences). Band density was measured using ImageQuant software.

### Immunoprecipitation and Western blot

Protein extraction of extracellular-enriched fraction and Western blot analysis for Aβ peptides was performed as described previously [13] with some modifications. 500 µg of protein per brain sample were incubated with NU-1 antibody in RIPA buffer (10 mM Tris-HCl (pH 7.4), 100 mM NaCl, 1 mM EDTA, 1 mM EGTA, 1% Triton X-100, 10% glycerol, 0.1% SDS, 0.5% deoxycholate, 1 mM PMSF, Leupeptin (3 µg/ml) and Aprotinin (5 µg/ml)) at 4°C for 1 h, followed by incubation with 40 µl of Protein A/G Plus agarose (Santa Cruz Biotechnology) at 4°C for overnight. Precipitants were washed four times with ice-cold RIPA buffer, and reconstituted with 24 µl of sample buffer, then incubated at 95°C for 3 min. 12 µl of immunoprecipitated sample from the brain or 20 µg of protein from microglia was electrophoresised on 16% SDS-polyacrylamide Tris-Tricine gel [43]. Proteins were transferred to 0.2 µm PVDF membrane (Bio-Rad). Membrane was blocked in 5% skim milk or 2% BSA/TBST (Tris-Buffered Saline-Tween 20), and incubated with pan-Aβ monoclonal antibody (6E10) or biotinylated 6E10 (Signet), followed by incubation with HRP-conjugated anti-mouse IgG (Jackson ImmunoResearch Laboratories) or HRP-conjugated streptavidin (Sigma). Blots were developed with West Pico supersignal chemiluminescent substrate (Pierce). Band luminescent intensities were quantified by ImageJ (NIH shareware program).

### Immunohistochemistry

Immunohistochemistry was performed as described previously [9], [27]. Briefly, mice were euthanized with isoflurane and perfused transcardially with 25 ml of ice cold PBS. The brains were rapidly removed, immersed in freshly depolymerized 4% paraformaldehyde for 48 h, and cryoprotected by successive 24 h immersions in 15% and 30% sucrose in 1× PBS immediately before sectioning. Fixed, cryoprotected brains were frozen and sectioned in the horizontal plane using a Cryostat (Leica), with sections collected serially. Immunohistochemistry was performed using specific antibodies to identify astrocyte (GFAP, rabbit polyclonal, 1∶2000, DAKO), microglia (IBA1, rabbit polyclonal, 1∶1000, Wako), and Aβ oligomer (NU-1, mouse monoclonal, 1 µg/ml [25]). Immunodetection was visualized using Envision Plus (DAKO) with 3,3′-diaminobenzidine (Vector Laboratories) for Aβ oligomer staining. For immunofluorescence, Alexa Fluor®488-conjugated anti-rabbit IgG (H+L) and Alexa Fluor®568-conjugated anti-mouse IgG (H+L) (Invitrogen) were used as secondary antibodies. Images were captured with a digital camera (DP71, Olympus) attached to a Nikon Eclipse TE300 inverted microscope using DP Controller and DP Manager (Olympus).

### Aβ aggregation assay

Primary cultured mouse microglia were prepared from WT day 0 newborn pups as described [26], [27]. For microglia, 5×10^4^ cells per well (in 96-well plate for immunofluorescence and microplate reading), or 2×10^5^ cells per well (in 24-well plate for biochemistry) were seeded for 1 week in Dulbecco's modified eagle medium (DMEM) supplemented with heat-inactivated 10% fetal bovine serum (FBS), 50 µg/ml penicillin/streptomycin (all from Invitrogen) and M-CSF. Aβ1−42 (Αβ42) peptide was commercially purchased (Invitrogen), and monomeric Aβ peptide was prepared according to manufacturer's instruction. Briefly, solid Aβ42 peptide was dissolved in cold hexafluoro-2-propanol (HFIP) and incubated at room temperature for 1 h. The HFIP was then removed by evaporation, and the resulting peptide was stored as a film at −20°C. The resulting film was dissolved in anhydrous DMSO with an Aβ42 final concentration of 250 µM, and then diluted to 1 or 10 µM in phenol red-free DMEM supplemented with heat-inactivated 10% FBS, 50 µg/ml penicillin/streptomycin (all from Invitrogen) and M-CSF. After pre-incubation with recombinant mouse CCL2 (10 ng/ml, R&D System), microglia were incubated without Aβ, with Aβ, with Aβ and CCL2 (10 ng/ml), with Aβ and TNF-α (10 ng/ml, R&D System), with Aβ and anti-CCL2 neutralizing antibody (1 ug/ml, R&D System), or with Aβ, CCL2 and anti-CCL2 neutralizing antibody for 1 h, then fixed with freshly depolymerized 4% paraformaldehyde for 15 min for immunofluorescence. Standard immunofluorescence was performed using Aβ oligomer (NU-2 monoclonal, 1 µg/ml [25]), and Alexa Fluor®488-conjugated anti-mouse IgG (H+L) (Invitrogen) as secondary antibody. Finally, cells were counterstained with Hoechst 33342 (1∶1000, Invitrogen) and fluorescent intensities measured by SpectraMAX M5 microplate reader (Molecular Devices) at excitation and emission wavelengths (Ex/Em) of 488 nm/519 nm for Alexa 488 and 350 nm/461 nm for Hoechst 33342.

### Statistics

All data were normally distributed and presented as mean values±standard errors of mean (SEM). In case of multiple mean comparisons, the data were analyzed by analysis of variances (ANOVA), followed by Newman-Keuls (for one-way ANOVA) or Bonferroni (for two-way ANOVA) multiple comparison test using statistics software (Prism 4.0, Graphpad Software, Inc.). In case of single mean comparison, data were analyzed by Student's *t*-test. p value of less than 0.05 was regarded as significant difference.

## Supporting Information

Figure S1 Balance beam, body weight, and swimming speed. Animals tested in Fig. 1 were examined using the balance beam with the average number of crossed segments per 3 minutes per animal shown for 2–3 (A) or 8–9 (B) months of age. The frequency of falling per group is shown in parenthesis. Average body weight of each group at 2–3 (C) or 8–9 (D) months of age, and average swimming speed at open field at 2–3 (E) or 8–9 (F) months of age were also tested. The average relative speed unit was determined by post-image acquisition analysis of actual pixels moved per second during the 60-second test. The numbers of mice tested were the same as Fig. 1 for all ages. * or # denotes p<0.05 versus WT or CCL2 group of the same age as determined by ANOVA and Newman-Keuls *post-hoc*.
(0.88 MB TIF)

Figure S2 CCL2 levels in the hippocampus at 5–6 months of age. The hippocampus of APP/CCL2, APP, CCL2, and WT mice at 5–6 months of ages (n = 6) were dissected and subjected for protein extraction in solubilization buffer. Murine CCL2 protein levels were determined using mouse CCL2 ELISA as described in the Material and Methods, respectively. *** denotes p<0.001 versus APP or CCL2 as determined by ANOVA and Newman-Keuls *post hoc*.
(0.80 MB TIF)
