# Supplementary figures and images for: CCL2 Accelerates Microglia-Mediated Aβ Oligomer Formation and Progression of Neurocognitive Dysfunction
Source: PLoS One. 2009 Jul 10;4(7):e6197. doi: 10.1371/journal.pone.0006197 (PMC2703798; doi:10.1371/journal.pone.0006197)

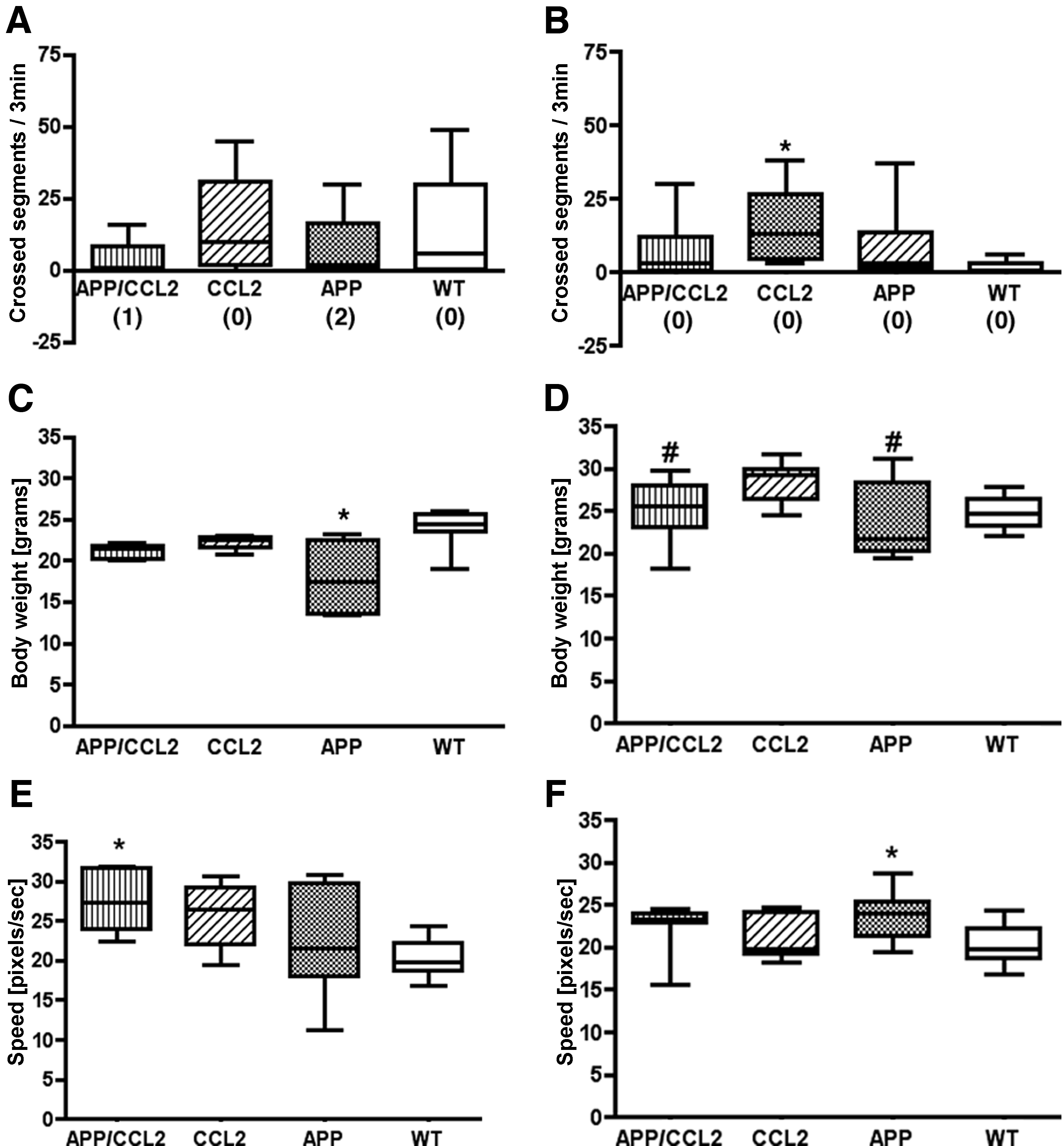

Supplement: Figure S1 — Balance beam, body weight, and swimming speed. Animals tested in Fig. 1 were examined using the balance beam with the average number of crossed segments per 3 minutes per animal shown for 2–3 (A) or 8–9 (B) months of age. The frequency of falling per group is shown in parenthesis. Average body weight of each group at 2–3 (C) or 8–9 (D) months of age, and average swimming speed at open field at 2–3 (E) or 8–9 (F) months of age were also tested. The average relative speed unit was determined by post-image acquisition analysis of actual pixels moved per second during the 60-second test. The numbers of mice tested were the same as Fig. 1 for all ages. * or # denotes p<0.05 versus WT or CCL2 group of the same age as determined by ANOVA and Newman-Keuls post-hoc. (0.88 MB TIF) [file pone.0006197.s001.tif]

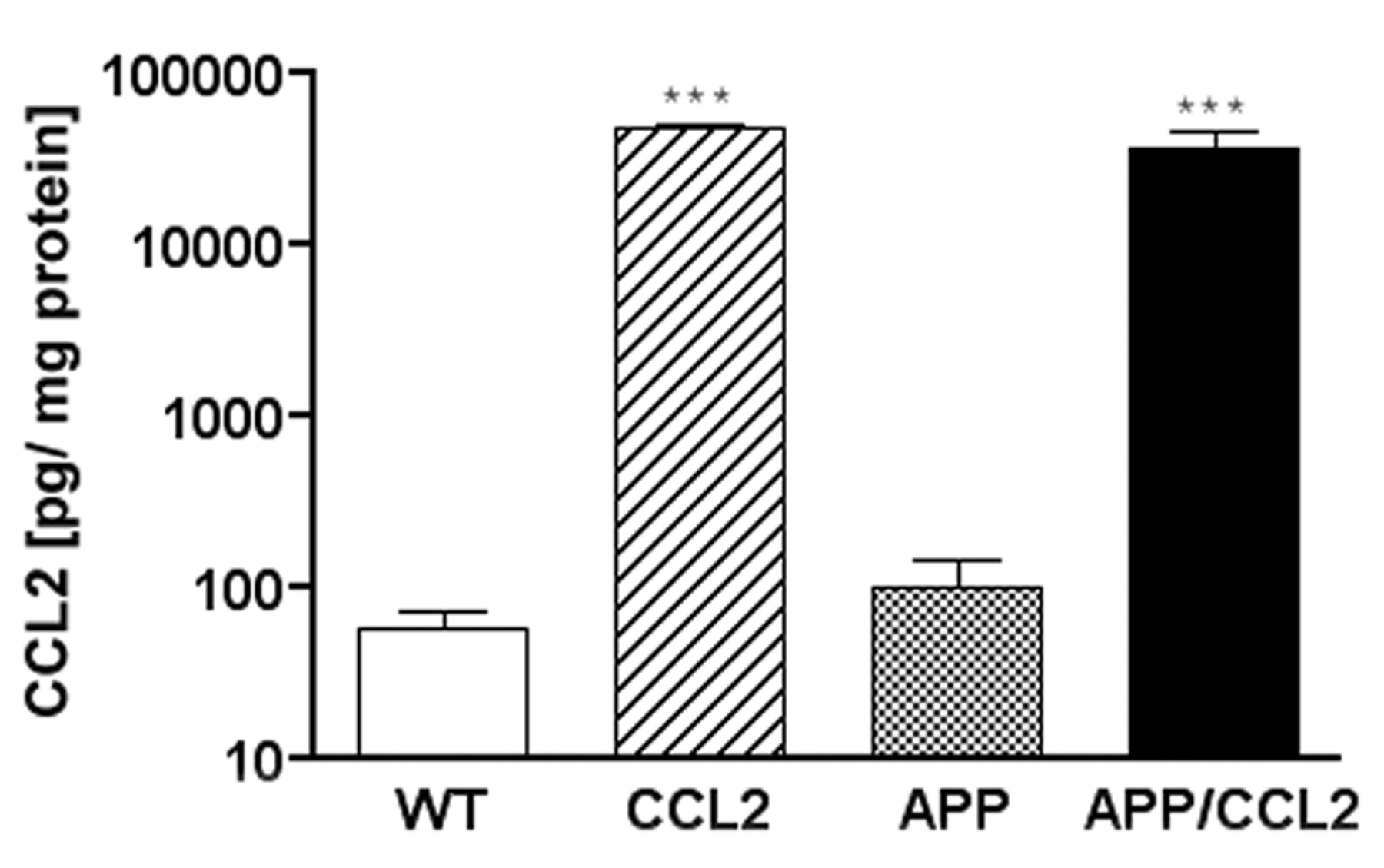

Supplement: Figure S2 — CCL2 levels in the hippocampus at 5–6 months of age. The hippocampus of APP/CCL2, APP, CCL2, and WT mice at 5–6 months of ages (n = 6) were dissected and subjected for protein extraction in solubilization buffer. Murine CCL2 protein levels were determined using mouse CCL2 ELISA as described in the Material and Methods, respectively. *** denotes p<0.001 versus APP or CCL2 as determined by ANOVA and Newman-Keuls post hoc. (0.80 MB TIF) [file pone.0006197.s002.tif]
